# Four Degrees of Separation: Social Contacts and Health Providers Influence the Steps to Final Diagnosis of Active Tuberculosis Patients in Urban Uganda

**DOI:** 10.1186/s12879-015-1084-8

**Published:** 2015-08-21

**Authors:** Juliet N. Sekandi, Sarah Zalwango, Leonardo Martinez, Andreas Handel, Robert Kakaire, Allan K. Nkwata, Amara E. Ezeamama, Noah Kiwanuka, Christopher C. Whalen

**Affiliations:** Department of Epidemiology and Biostatistics, College of Public Health, University of Georgia, B.S. Miller Hall, 101 Buck Rd, Athens, GA 30602 USA; Makerere University College of Health Sciences, School of Public Health, Kampala, Uganda; Department of Health Services, Kampala City Council Authority, Kampala, Uganda; Makerere University College of Health Sciences, School of Medicine, National TB Treatment Center, Mulago Referral and Teaching Hospital, Mulago, Uganda

## Abstract

**Background:**

Delay in tuberculosis (TB) diagnosis adversely affects patients’ outcomes and prolongs transmission in the community. The influence of social contacts on steps taken by active pulmonary TB patients to seek a diagnosis has not been well examined.

**Methods:**

A retrospective study design was use to enroll TB patients on treatment for 3 months or less and aged ≥18 years from 3 public clinics in Kampala, Uganda, from March to July 2014. Social network analysis was used to collect information about social contacts and health providers visited by patients to measure the number of steps and time between onset of symptoms and final diagnosis of TB.

**Results:**

Of 294 TB patients, 58 % were male and median age was 30 (IQR: 24–38) years. The median number of steps was 4 (IQR: 3, 7) corresponding to 70 (IQR: 28,140) days to diagnosis. New patients had more steps and time to diagnosis compared retreatment patients (5 vs. 3, *P* < 0.0001; 84 vs. 46 days *P* < 0.0001). Fifty-eight percent of patients first contacted persons in their social network. The first step to initiate seeking care accounted for 41 % of the patients’ time to diagnosis while visits to non-TB providers and TB providers (without a TB diagnosis) accounted for 34 % and 11 % respectively. New TB patients vs. retreatment (HR: 0.66, 95 % CI; 1.11, 1.99), those who first contacted a non-TB health provider vs. contacting social network (HR: 0.72 95 % CI; 0.55, 0.95) and HIV seronegative vs. seropositive patients (HR: 0.70, 95 % CI; 0.53, 0.92) had a significantly lower likelihood of a timely final diagnosis.

**Conclusions:**

There were four degrees of separation between the onset of symptoms in a TB patient and a final diagnosis. Both social and provider networks of patients influenced the diagnostic pathways. Most delays occurred in the first step which represents decisions to seek help, and through interactions with non-TB health providers. TB control programs should strengthen education and active screening in the community and in health care settings to ensure timely diagnosis of TB.

**Electronic supplementary material:**

The online version of this article (doi:10.1186/s12879-015-1084-8) contains supplementary material, which is available to authorized users.

## Background

Delays in the diagnosis of active pulmonary tuberculosis (TB) present a major obstacle to TB control [1–3] because these delays can perpetuate transmission of *M. tuberculosis* in the community and health care settings. Most transmission of *M. tuberculosis* occurs during the infectious period between the onset of cough and the initiation of treatment and is influenced by the contact patients make with susceptible individuals [1]. Ideally, persons who experience TB symptoms should be promptly identified, diagnosed, and initiated on effective treatment, but this is far from routine practice in developing countries, including Uganda [4–7]. Previous systematic reviews have reported widely varying diagnostic delays in low- and high-income settings [8, 9]. Some factors that have been commonly reported to influence care-seeking from the health system include low education, old age, poverty, smoking and alcohol use, lack of access to health facilities and HIV infection [1, 9–14]. However, the broader social context in which delays in TB diagnosis occur has not been fully explored in the published literature.

We posit that TB patients are members of community networks that include interactions with social contacts and health providers. Before the diagnosis of TB is made, patients must navigate their personal networks until they reach a health care provider who can make the proper diagnosis and initiate effective treatment. Previous studies mainly focus on interactions with the health providers and show that patients are sometimes trapped in vicious cycles of repeated visits to the same health providers, especially private clinics and drug stores who do not provide specialized TB services [1, 2, 6, 9–11, 13]. But, far fewer studies have systematically examined the patients’ patterns of steps when seeking pre-diagnosis care in the informal and formal health system [10, 15]. Moreover, none of these studies evaluate the time spent within the patients’ social network before contacting health providers. In this study, we used network analysis to estimate the number of steps to, or degrees of separation from, diagnosis in new and retreatment TB patients, and then determined the factors associated with the steps and time to diagnosis.

## Methods

### Study design, setting and population

We conducted a retrospective cohort study among TB patients attending three public health clinics located in Kampala, Uganda from March to July 2014. The study population comprised patients who were diagnosed with active TB between January 1 and July 31, 2014. This constituted a prevalent cohort from which participants were recruited at variable times after diagnosis and retrospective information on time of seeking care before the final diagnosis was collected during interviews; this approach was deemed a suitable alternative to a prospective cohort study [16]. A completed STROBE check list is also provided in additional supplementary file 1: S1. The study clinic sites are part of the government–funded, public health system in Kampala that provides free TB diagnosis and treatment services. Consenting adult patients were eligible for the study if they were 18 years or older, had active pulmonary TB, and had initiated TB treatment within three months on the day of they were approached to enroll in the study. Patients were excluded if they had extra-pulmonary TB, did not speak English or Luganda, or were too ill to complete interviews. A completed STROBE check list is also provided in Additional file 1. During recruitment, 300 active pulmonary TB patients were identified in pharmacy waiting areas of medical clinics and then screened for study eligibility; six patients were excluded because of inconsistencies in reported time to diagnosis, leaving 294 study patients.

A new active pulmonary TB patient was defined as a patient that had a confirmed bacteriologic or radiologically confirmed diagnosis of TB for the first time. A retreatment case was defined as a patient who had a confirmed TB diagnosis with a previous history of TB disease and a medical record of completion of anti-TB treatment and cure.

### Conceptual model for diagnostic pathways and operational definitions

**Fig. 1:**
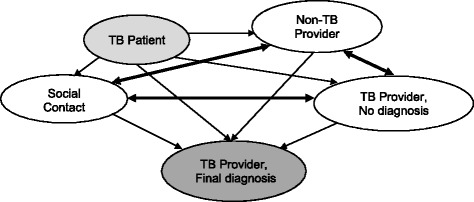
Conceptual Model of Social and Provider Contacts in TB Patients’ Diagnostic Pathway. Legend: The TB patient (lighter grey) initiates movement through the network. Solid lines are directional and indicate the types of movement between contacts in the network; heavier lines have double arrows representing bidirectional movement allowing for iterative paths and loops in the networks. The TB provider making the final diagnosis (darker grey) represents the absorbing step for all movements

**Fig. 2:**
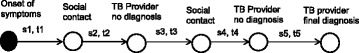
Illustration of a Realized Pathway to Final TB Diagnosis. Legend: Solid line represents the linear representation of the path through the diagnostic network. Non-solid circles represent the contact points, or nodes, of the network leading to diagnosis. The interval between each node is characterized by a step number (s) and a time interval (t). The total steps to diagnosis is s1 + s2 + s3 + s4 + s5. Total time to diagnosis is t1 + t2 + t3 + t4 + t5

The diagnostic pathway was defined as the health care seeking continuum from the initial self-recognition of TB symptoms to the final diagnosis. We conceptualized that each active pulmonary TB patient is part of a unique network that consists of social contacts and health providers (Fig. 1). When a patient develops symptoms, s/he may turn to any member of their network for advice or help. That first interaction may lead to the next contact, and a series of others, until the patient arrives at a health provider who makes the final diagnosis. Since networks are complex, there are many potential pathways through a network leading to a final diagnosis. In the end, each TB patient realizes his or her own path, or sequence of contacts, through the network until diagnosis is made (Fig. 2). The realized pathway may or may not be unique to any given patient.

We decomposed the diagnostic pathways into a series of steps or degrees of separation. A step was defined as a contact, or interaction, between a person or place in the course of seeking care for symptoms (Fig. 2). Each step was associated with a period of time; the time to diagnosis was defined as the total time from onset of symptoms to TB diagnosis.

We partitioned the steps into two categories: steps to social contacts and steps to health care providers. A social contact was defined as any person in the community who is not a health provider, such as family members, relatives, friends, or workmates. A health provider was defined as any person or place that offers any form of treatment or advice to relieve symptoms [1, 11]. The provider network was further partitioned into two groups: TB providers and non-TB providers. TB providers were those who provided standard, specialized TB diagnostic and therapeutic services as designated by the Uganda Ministry of Health; they included government hospitals, private hospitals and government health centres. The TB providers with no diagnosis are those who failed to make the diagnosis of TB during a given contact. The non-TB providers were health care providers who are not designated to provide specialized TB services; they included private clinics, drug stores, herbal healers, and village health workers.

### Study outcome

English version of questionnaire provided as Additional file 2. The main outcome of the study was the count of steps to TB diagnosis and the secondary outcome was the time to diagnosis. For each patient, the number of steps was the number of sequential pairs in a patient’s diagnostic path (n – 1, where n is the number of contacts along the path). In counting the number of steps, we allowed for patients to return to the same social contact or health provider on multiple occasions; each listed contact was considered an independent step.

### Data collection and management

Data were collected in face-to-face interviews by trained interviewers using a structured questionnaire that was developed by a team of physicians with expertise in TB and epidemiologists. The questionnaire was translated in Luganda language (local dialect). We tested the questionnaire for accuracy, comprehension, and consistency of responses in a pilot, the overall results were satisfactory. The questionnaire items included socio-demographics, previous TB history, previous TB treatment, knowledge about TB-related symptoms, HIV serostatus, time of diagnosis, time of onset of symptoms and duration of symptoms (English version of questionnaire provided as additional supplementary file 2: S2). Retrospective information about the patients’ care seeking experiences before final diagnosis was reconstructed and the time intervals that elapsed. To reduce recall bias, the interviewers used a combination of name and location generators, or standard prompts, and recent time-frames to help participants recall contacts. These prompts included, for example, questions about family, friends, neighbors, classmates, work associates, recent travel and other prompts appropriate to the interview. Data were collected using standardized teleforms and scanned into a database using optical scanning software (TeleForms®).

### Statistical analysis

Following a descriptive analysis, we performed a sequence analysis by reconstructing the steps, and time interval between the steps, according to the type of step. We examined four types of steps: self to any contact in the network as the initial step; social contact to any other contact in network except self; non-TB provider to any type of contact; and TB provider, no diagnosis, to any type of contact. The sequence of patterns were compared by disease type (i.e., new or recurrent) and HIV status. We constructed time-to-event data in a retrospective manner as has been done in previous studies [16]. We added up the steps and time in days between onset or self-recognition of symptoms up to the date of TB diagnosis. Since all enrolled patients had a final TB diagnosis event, there was no censoring. We performed a series of Cox proportional hazards regression models to identify factors that influenced the steps, or time, to TB diagnosis. The covariates included in the model were age, sex, marital status, employment, TB treatment category and HIV status and place or person first contact for help. The proportional hazard assumptions of the Cox model were met; hazards ratios with 95 % confidence intervals are presented. The level of significance was *p* < 0.05 for all analyses. Stata version 12.1 (Statacorp, College Station, Texas) and R 3.1.3 software were used for analysis.

### Ethical considerations

Written informed consent was obtained from all eligible TB patients. The study was approved institutional review boards at the University of Georgia, Makerere University School of Public Health and the Uganda National Council for Science and Technology.

## Results

Of the 294 study patients, 224 (76 %) were new and 70 (24 %) were retreatment patients (Table 1). The median age of the patients was 30 years, 37 % were young adults aged 25–34 years. More than half of the patients were male and 59 % resided in the subdivisions of Kampala City. Most of the patients were employed, and the median monthly income was equivalent to US $80. About one-third of all enrolled patients were HIV seropositive and 4.6 % did not know their HIV status, 13 % currently smoked, and 80 % owned cellphones.

**Table 1:** Baseline characteristics of 294 participants in Kampala, Uganda, April–July 2014

Characteristics	Frequency	Percent
Median Age (IQR), yrs	30 (24–38)	NA
Age groups		
18–24	74	25.0
25–34	108	37.0
35–44	71	24.3
≥45	41	13.7
Sex		
Female	124	41.3
Male	170	58.7
Marital status		
Never married	103	35.0
Currently married	106	36.7
Previously married	85	28.3
Division of Residence		
Rubaga	26	9.0
Nakawa	12	4.0
Central	27	9.7
Kawempe	76	26.0
Makindye	31	10.3
Other^a^	122	41.0
Employed^c^		
Yes	244	83.3
No	50	16.7
Median Monthly Income, US$ (IQR)^b^	80 (40–160)	NA
TB Treatment Category		
New	224	76.1
Retreatment	70	23.9
HIV Status		
Positive	96	32.7
Negative	184	62.7
Don’t know	14	4.6
Smoking Status		
Current Smoker	34	13.0
Previous Smoker	41	15.6
Non Smoker	190	71.4
Missing	29	-
Cellphone Ownership		
Yes	237	81.0
No	57	19.0

^a^ *Others* = Towns outside of the 5 divisions of Kampala district

^b^Income in Uganda Shillings (1US$ ~ Ushs 2500)

^c^Includes both formal and informal employment

The median number of steps to diagnosis taken by 294 patients was 4 (IQR: 3,7), corresponding to 70 (IQR: 28,140) days to diagnosis. The steps to diagnosis varied by TB treatment category; new TB patients had a greater median number of steps to diagnosis compared to retreatment patients (5 vs. 3, *p* < 0.0001; Table 2). New patients also had a longer median time to diagnosis compared to retreatment patient (84 vs. 46 days, *p* < 0.0001; HIV sero negative patients had the same median number of steps as HIV seropositive patients, but had a longer median time to diagnosis (Table 2).

**Table 2:** Steps and time to diagnosis by patients’ clinical characteristics

Characteristics	Total number of patients	Steps to diagnosis (Median, IQR)	*P*-value	Days to diagnosis (Median, IQR)	*P*-value
Overall	294	4 (3,7)		70 (28,140)	
TB treatment category					
New	224	5 (3,8)		84 (42,168)	
Retreatment	70	3 (3,4)	<0.0001^a^	46 (26,84)	<0.0001^a^
HIV Status					
Positive	96	4 (3,6)		17 (6,60)	
Negative	184	4 (3,8)		42 (16,118)	
Unknown	14	5 (4,7)	0.081^b^	59 (20,90)	0.0004^b^

^a^Wilcoxon Rank-sum test: *p*-value for comparison of two-sample medians

^b^Kruskal-Wallis rank test *p*-value for comparison of two or more sample medians

**Fig. 3:**
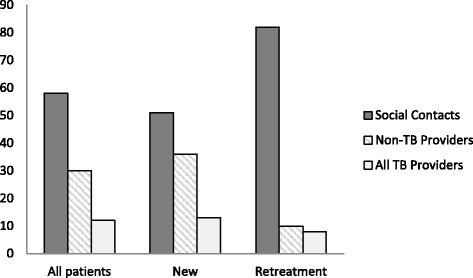
Percent of TB Patients’ First Contact by TB Treatment Category

**Fig. 4:**
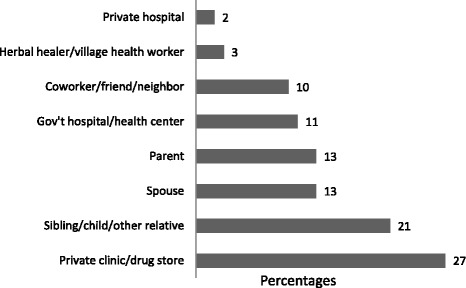
Percent Distribution of First Contact Place or Person. Legend: Gov’t hospital, health center and private hospital = All TB Provider. Herbal healer, private clinics, drugs stores, village health worker = Non-TB providers. Parent, spouse, siblings, adult child, other relative, friend, co-worker, neighbor = Social contacts

The first point of contact was most often with a member of the patients’ social network (Fig. 3); 58 % of all patients initially approached a family member, relative, friend or co-worker for advice or help (Fig. 4). The next most common point of first contact was the non-TB provider networks; 30 % of patients contacted non-TB providers which included private clinics, drug stores, herbal healers or village health care workers. Of these points of contacts, private clinics/drug stores were the most common (Fig. 4). TB care providers were least likely to be visited as initial contact. When we stratified by new or retreatment TB category, the results were similar in direction.

### Patterns of steps and time to diagnosis

The 294 patients made 1555 step pairs altogether within their social and provider networks to reach a diagnosis (Table 3). Most (45 %) of the steps were from non-TB providers to any other contact, followed by steps from social contacts (23 %) to contacts in the network. The cumulative person-days spent by patients in all the steps until diagnosis were 32,011 days and the median person-days spent in any step interval was 14 days (IQR: 3, 27). The first step taken after symptom recognition to any network member comprised 19 % of the total steps, took 13,220 person-days (median: 29 days, IQR; 16, 58), accounting for 41 % of the total care seeking person-days. Steps from non-TB providers to any network member comprised 45 % of the total steps, took 10,872 person-days (median = 14 days: IQR; 7, 30), accounting for 34 % of the care-seeking person-time. Overall, when considering component of each step pattern, 64 % of the steps and 75 % of the time before diagnosis were spent deciding to seek help or following care advice from non-TB providers. When stratifying by HIV serostatus of the TB patient, a similar pattern is found. When stratifying by type of TB, retreatment patients still delay in seeking care, but spent less time and fewer steps with non-TB providers.

**Table 3:** Step patterns and person- days in diagnostic pathway by social and health provider contacts among 294 TB patients

Step patterns					
From	To	Steps pairs^c^ (%)	Total person- days^d^ (%)	Median person-days^e^ [IQR]	Number of patients in step^f^
All steps		1,555	32,011	14 (3,27)	294
Self	Social contact	171 (11)	7249 (23)	28 (14,53)	171
Self	Non-TB provider	87 (5.4)	4009 (12)	29 (16,62)	87
Self	TB provider, no diagnosis^b^	27 (2)	1353 (4)	54 (18,69)	27
Self	TB provider, final diagnosis^a^	9 (0.6)	609 (2)	68 (21,91)	9
Self	Any contact	294 (19)	13,220 (41)	29 (16,58)	294
Social contact	Social contact	30 (2.4)	515 (2)	14 (2,19)	26
Social contact	Non-TB provider	171 (11)	2364 (7)	14 (2,14)	141
Social contact	TB provider, no diagnosis^b^	77 (5)	1123 (4)	7 (1,14)	75
Social contact	TB provider, final diagnosis^a^	73 (4.6)	456 (1)	2 (1,14)	73
Social contact	Any contact	351 (23)	4458 (14)	7 (7,77)	315
Non-TB provider	Social contact	135 (9)	1746 (5)	7 (2,14)	92
Non-TB provider	Non-TB provider	405 (26)	6801 (21)	14 (7,21)	139
Non-TB provider	TB provider, no diagnosis^b^	43 (3)	825 (3)	14 (3,26)	41
Non-TB provider	TB provider, final diagnosis^a^	118 (7)	1500 (5)	7 (1,14)	118
Non-TB provider	Any contact	701 (45)	10,872 (34)	14 (7,30)	390
TB provider, no diagnosis	Social contact	15 (0.9)	260 (0.8)	14 (3,22)	14
TB provider, no diagnosis	Non-TB Provider	38 (2.1)	696 (2.2)	11 (4,30)	37
TB provider, no diagnosis	TB provider, no diagnosis^b^	62 (4)	1551 (5)	14 (5,30)	36
TB provider, no diagnosis	TB provider, final diagnosis^a^	94 (6)	952 (3)	3 (1,14)	94
TB provider, no diagnosis	Any contact	209 (13)	3459 (11)	7 (3,17)	181
All steps		1,555	32,011	14 (3,27)	294

*Self* = Symptom onset or recognition

*TB Provider* = Gov’t hospital, health center and private hospital

*Non-TB providers* = Herbal healer, private clinics, drugs stores, village health worker

*Social Contact* = Parent, spouse, siblings, adult child, other relative, friend, co-worker, neighbor

^a^TB provider where final diagnosis took place; all patients performed this step only once

^b^TB provider, no diagnosis where a TB diagnosis was not made in the step

^c^Total step pairs capture patients’ movements between two contact points along their pathway

^d^Total time represents cumulative person-days spent in a given step pair by all patients who performed it

^e^Median person-days spent by patients who performed each step pair

^f^Number of patients taking steps, this may exceed *N* = 294 because some patients performed the same step pairs more than once

In a univariate analysis, the median steps to diagnosis showed significant relationships with marital status, employment, HIV status, TB treatment category, and first contact for help (Table 4). In the multivariable Cox regression analysis of steps to diagnosis, 44 % of new patients had fewer steps to diagnosis compared with the retreatment patients after adjusting for age, HIV status, and the first contact within a network (HR: 0.66, Table 4). When patients first contacted a non-TB, they were less likely to have fewer steps to diagnosis of TB than if first contact was in the social network (HR: 0.72, Table 4). In an analysis using time to diagnosis as the main outcome, HIV status was also a significant factor, showing that HIV seronegative patients were 30 % less likely to reach a final diagnosis compared to HIV seropositive patients (HR: 0.70, Table 4). Similar results were obtained in magnitude and direction of association with TB treatment category.

**Table 4:** Relative likelihood of TB final diagnosis expressed as hazard ratios using cox regression analysis

		Steps to diagnosis		Days to diagnosis	
Characteristics	Total (%)	Unadjusted HR (95 % CI)	Adjusted HR (95 % CI)	Unadjusted HR (95 % CI)	Adjusted HR (95 % CI)
Age category					
18–24	74 (25)	1.00	1.00	1.00	1.00
25–34	108 (37)	0.89 (0.66,1.19)	0.82 (0.60,1.11)	0.97 (0.72,1.30)	0.81 (0.59,1.10)
35–44	71 (24.3)	0.91 (0.66,1.26)	0.72 (0.50,1.03)	0.96 (0.69,1.34)	0.88 (0.59,1.16)
≥45	41 (13.7)	1.48 (1.11,2.16)	1.37 (0.92,2.04)	1.70 (1.15,2.50)	1.14 (0.77,1.68)
Sex					
Female	124 (41.3)	1.00	-	1.00	-
Male	170 (58.7)	1.07 (0.85.1.35)		1.19 (0.94.1.51)	
Marital status					
Never married	103 (35)	1.00	-	1.00	-
Currently married	106 (36.7)	0.83 (0.64,1.09)		0.76 (0.58,1.00)	
Previously married	85 (28.3)	1.08 (0.81,1.44)		0.97 (0.74,1.33)	
Employment					
Yes	244 (83.3)	1.00	-	1.00	-
No	50 (16.7)	0.84 (0.62,1.14)		0.87 (0.64,1.19)	
Mobile phone ownership					
Yes	237 (81)	1.00	-	1.00	-
No	57 (19)	1.16 (0.87,1.55)		1.07 (0.79,1.43)	
TB treatment category					
Retreatment	70 (23.7)	1.00	1.00	1.00	1.00
New	224 (76.3)	0.59 (0.45,0.78)*	0.66 (0.49,0.88)*	0.62 (0.47,0.81)*	0.70 (0.52–0.93)*
HIV status					
Positive	96 (32.7)	1.00	1.00	1.00	1.00
Negative	184 (62.7)	0.84 (0.66,1.11)	0.88 (0.67–1.15)	0.77 (0.60–0.96)	0.70 (0.53–0.92)*
Don’t know	14 (4.6)	0.85 (0.49,1.49)	0.95 (0.54–1.68)	0.85 (0.47–1.52)	1.23 (0.68–2.33)
First contacted place/person					
Social	171 (58)	1.00	1.00	1.00	1.00
TB care provider	36 (12)	0.99 (0.69,1.42)	1.08 (0.75,1.57)	0.88 (0.62,1.27)	0.89 (0.62,1.29)
Non-TB care provider	87 (30)	0.65 (0.50,0.85)*	0.72 (0.55,0.95)*	0.71 (0.55,0.92)*	0.50 (0.40,0.70)*

*significant at *p* < 0.05

## Discussion

In this retrospective study, we found that on average, active pulmonary TB patients took four steps to reach final diagnosis; in other words, there were four degrees of separation between a the onset of symptoms in a TB patient and final diagnosis. Although the average time to diagnosis was 70 days, the cumulative time until diagnosis across all patients was 32,011 person-days, or nearly 88 person-years. These delays may affect extent of disease at presentation and outcomes of treatment. Moreover, they create numerous opportunities for disease transmission within the networks of patients. While previous studies have examined delays in the health care system using time as the main metric [1, 4, 9, 14], we added a new way to measure delays in diagnosis using the number of steps taken within the patient’s provider and social network. The dimension of information about the persons or places that patients contact as they traverse their network in seeking care could perhaps help expand the scope of interventions to include community members.

The underlying premise of this study is that patients must navigate through their own social and provider network to arrive at a proper diagnosis of TB. There is no standard threshold for steps to diagnosis, but, ideally patients should take one step to seek care at a specialized TB diagnostic center once they recognize suspicious symptoms. Since patients may be unaware of the cause of their symptoms, they may seek care first through a primary care provider who then refers to a clinic with specialize services for TB. In this case, two steps within the provider network are reasonable. In our study, patients took two additional steps beyond this ideal to reach a diagnosis, but the range was wide as half of the patients took more than four steps. A recent systematic review from studies done in India showed an average of 2.7 steps while considering only health care providers [9]. Steps alone, however, are not sufficient to explain the risk to patients in the community. The time in each step is also important. As shown in our study, an average step length of 2 weeks reflects substantial time delays as patients make new contact within their network.

The use of network analysis provides additional insights above and beyond the use of time alone because it provides details about the nature of the contacts made during the time leading up to diagnosis. These details shed light on how and where transmission of *M. tuberculosis* may occur, which may, in turn, inform ways of interrupting transmission.

We found that the greatest delay in care-seeking occurred with the first step, which is the recognition by the patient that the symptoms require attention. In fact, 41 % of the total time seeking care was spent in this first step. This delay may be due to a lack of knowledge about health, stigma with TB, a low perception of TB risk, or seriousness of symptoms [17, 18]. When patients did initiate steps to seek care, they most often sought advice from members in their immediate social network, including spouses, siblings, parents, and occasionally co-workers. It was surprising that when patients first consulted members of their social network, they were able to reach a correct diagnosis earlier than if they first consulted a non-TB provider. These findings illustrate the value of family and social support in avoiding unnecessary delays in TB diagnosis [11, 19, 20].

Apart from the initial delay in seeking care, the steps involving non-TB providers also introduced substantive delays in the diagnosis of TB. Steps involving non-TB providers comprised 34 % of the time before diagnosis, whereas steps taken from a TB-TB provider, with no diagnosis to any member of the network comprised 11 % of the time to diagnosis. Previous studies have shown that patients may spend much of their care seeking time cycling repeatedly in private clinics and drug stores where they were not likely to be diagnosed [1, 9, 10]. Surprisingly, we also found that even when patients contacted specialized TB providers, additional steps were taken before a diagnosis was made. This may point to existing gaps in the overall quality of TB services even in specialized care centers [21, 22].

The findings of this study have important implications for medicine and public health. From the medical perspective, delays in diagnosis affect extent of disease at diagnosis and treatment outcomes [23]. From the public health perspective, delays in diagnosis prolong the infectious period of an index case, thereby prolonging the transmission in the community [3] and health-care settings [24–27].

The findings of our study suggest three targets for interventions that affect the time to TB diagnosis. First, the undiagnosed, infectious patients often delay seeking advice and care, even from family or friends. To reduce this delay, community education campaigns should be designed to increase knowledge about the signs and symptoms of TB. Mass media, trained community volunteers, and use of mobile technology have been shown to improve TB knowledge and awareness [28–30]. Additionally, barriers to health care access should be minimized by expanding TB services through periodic community screening integrated with routine primary health outreach programs [31]. Second, non-TB providers often perpetuate delays in diagnosis. Since they constitute an important first access point for care for patients, they should be optimally prepared to diagnose and treat patients, or refer symptomatic persons to specialized clinics. Non-TB providers should complete continuing medical education that emphasizes TB screening using standard algorithms in individuals with chronic cough. Incentives for proper practice may enhance early diagnosis in private care settings [31]. Finally, the specialized TB providers should be targeted with continuing medical education to strengthen their clinical ability to suspect, identify and evaluate symptomatic patients so as to avert missed opportunities for diagnosis TB [31, 32].

This study used the network approach to generate additional information about the influence of the patients’ social and provider contacts on TB patients’ diagnostic pathways. We used interview prompts to reconstruct patients’ experiences on sequence of steps and time hence generating retrospective data. However, our study findings are subject to limitations. The self-reported data about the places or persons contacted for help and the time before diagnosis are subject to recall bias. The potential effect of recall bias was minimized by excluding patients who had been diagnosed more than three months from the survey date. We assumed that each event was separate, but acknowledge that it is possible that contact events occurred concurrently leading to overestimation of the steps to diagnosis.

## Conclusions

In summary, there were four degrees of separation between onset of symptoms in a TB patient and a proper diagnosis. The social and provider networks of patients played a central role in determining the TB diagnostic pathways. The main delays in diagnosis occurred with the initial decision to seek advice or care, through interactions with non-TB providers. Additionally, there are missed opportunities for diagnosis at specialized TB centers. Future programs should strengthen TB education and active case finding in the community and in health care settings to ensure timely diagnosis of TB.

## Additional files

Additional file 1:
**STROBE Statement—checklist of items that should be included in reports of observational studies.** (PDF 97 kb) Additional file 2:
**English questionnaire TB Steps.** (PDF 28 kb)
