# Supplementary material for: Four Degrees of Separation: Social Contacts and Health Providers Influence the Steps to Final Diagnosis of Active Tuberculosis Patients in Urban Uganda
Source: BMC Infect Dis. 2015 Aug 21;15:361. doi: 10.1186/s12879-015-1084-8 (PMC4546132; doi:10.1186/s12879-015-1084-8)
Supplement: Additional file 2: — English questionnaire TB Steps. (PDF 28 kb) [file 12879_2015_1084_MOESM2_ESM.pdf]

28306

MAKERERE UNIVERSITY-UNIVERSITY OF GEORGIA RESEARCH COLLABORATION
COMMUNITY HEALTH AND SOCIAL NETWORKS OF TUBERCULOSIS
DIAGNOSTIC PATHWAY FORM_ENGLISH

Date of Interview ☐☐ / ☐☐ / 2 0 ☐☐
Day Month Year

STUDY ID# ☐☐☐

1. In which division do you live?
[Prompt] ☐☐
01=Rubaga 04=Kawempe
02=Nakawa 05=Makindye
03=Central 88=Other ☐

2. How old are you? ☐☐ [Yrs]

3. Sex ☐ Male ☐ Female

4. What is your marital status?
☐☐
01 = Single/Never married
02 = Married/Cohabiting
03 = Separated/divorced
04 = Widowed

5. Are you employed ? ☐ Yes ☐ No

6. What is your main occupation? Whether you earn or don't earn an income from it
Specify ☐

7. On average, how much money do you earn in a month from all you jobs?
Shs ☐☐☐☐☐☐☐

8. Is this your first TB episode?
☐ Yes ☐ No

9. When did you receive your current TB diagnosis?
☐☐ / ☐☐ / 2 0 ☐☐
Day Month Year

10. Where was your current TB diagnosed?
☐☐
01 = Private clinic
02= Private Hospital
03 = KCCA clinic
04= Gov't Hospital
05 =Gov't Health center
88= Other,specify ☐

11. For how long did you have your cough, or other symptoms, before you received this TB diagnosis?
Days ☐☐ Weeks ☐☐ Months ☐☐

12. For how long did you have these symptoms before you first sought advice from any person about what to do?
☐☐ ☐ Days ☐ Months ☐ Weeks ☐ Not Applicable
Code 99 if Not Applicable

13. For how long did you have your cough or other symptoms before first sought treatment from a health provider or facility?
☐☐ ☐ Days ☐ Months ☐ Weeks ☐ Not Applicable
Code 99 if Not Applicable

Knowledge Assessment

14. Please, tell me some of the symptoms of TB that you knew about before receiving your diagnosis? [***Do not prompt respondents***] Mark all that apply
☐ Cough >=2 weeks ☐ Coughing up blood
☐ Evening Fever ☐ None
☐ Weight Loss ☐ Other,Specify ☐
☐ Excessive Sweating

15. Did you think you had TB before you were diagnosed?
☐ Yes ☐ No

16. Did someone else, such as a family member or friend, express concern about your illness before your TB diagnosis was
☐ Yes ☐ No

17. Did you know that TB is spread from a person who has disease before receiving your current TB diagnosis?
☐ Yes ☐ No

HIV Status and Comobidities

18. What is your HIV status?
☐ Positive ☐ Negative ☐ Don't Know

19. If 'Positive", are you currently taking antiretroviral therapy?
☐ Yes ☐ No ☐ Not Applicable

20. Do you suffer from any chronic illnesses such as diabetes, hypertension or any other that require you to visit the health facility regularly?
☐ Non ☐ Hypertension
☐ Diabetes ☐ Other,Specify ☐

Cell Phone Ownership and Use

21. Do you own a cell phone? ☐ Yes ☐ No

22. If "No", do you share a cell phone ☐ Yes ☐ No

23. If "Yes" have you ever used a cell phone to contact a health provider for advice or help?
☐ Yes ☐ No ☐ Not Applicable

24. If "Yes" have you ever used a cell phone to contact family members or friends for advice or help about your health?
☐ Yes ☐ No

25. Did you use your cell phone in anyway to seek help for this current TB illness?
☐ Yes ☐ No

28306

DIAGNOSTIC PATHWAY FORM_ENGLISH

Part B

Date of Interview __ __ / __ __ / 2 0 __ __ (Day / Month / Year)

STUDY ID# __ __ __

26. **Preamble to be read by Interviewer:** Now, think back about when you first noticed your symptoms of TB. We are interested in knowing the people you talked to about your symptoms and the places you may have sought care after you started feeling ill. Please tell me about what you did, who you confided in about your illness, or where you went to seek help before you had the final diagnosis of TB. **Remember**, this could be a family member, relative, friend, co-worker or any health provider or facility. I also want to know if you talked to the same person or visited the same place for help more than once.[Interviewer: Be sure to document separately each time a person or place is mentioned by the patient even if they are the same]

Now to begin, please tell me the first person/place you approached when your symptoms began. Remember, this may be a family member or friend. [Collect and Record]..........Good, can you tell me the NEXT person or place after that? [Collect & Record] [**Continue** with this line of questioning, with appropriate prompting and explanation, until patient indicates the place of diagnosis].

Contacts	Contact Person or Place	Code	Household member	Referred by	Time btn contact	Unit for Time	Outcome
Contact 1					0 0	0 0	
Contact 2							
Contact 3							
Contact 4							
Contact 5							
Contact 6							
Contact 7							
Contact 8							
Contact 9							
Contact10							
Contact11							
Contact 12							
Contact 13							
Contact 14							

USE THE CODES BELOW TO COMPLETE TABLE ABOVE

Code for contact person or places visited ***QN: What person or place did you contact or visit for help/ treatment?***
01 = Herbal healer 02 = Drug store 03 = Gov't hospital 04 = Private hospital 05= Private clinic 06 = Gov't Heath center 07= Village health worker 08 = Spouse 09 = Parent 10= Brother/sister 11=Other relative 12=Co-worker 13 = Friend 14=Child 15= Neighbor 88=Other 99=Not applicable

Code for Household Member ***QN: Does the person listed live in the same household as the patient?***
01=Yes 02= No 99= Not applicable

Referred By: ***QN: Who referred you to [Name of the person/ place] (mention the person or name of place as listed in Qn.26 above)***
01 = Herbal healer 02 = Drug store 03 = Gov't hospital 04 = Private hospital 05= Private clinic 06 = Gov't Heath center 07 = Village health worker 08 = Spouse 09 = Parent 10= Brother/Sister 11=Other relative 12=Coworker 13 = Friend 14=Child 15= Neighbor 16= Self 88= Other 77= Uncertain

Units for Time: ***QN: How much time elapsed between persons contacted or places visited [Refer to list of contacts above]*** 01 = Days 02 = Weeks 03 = Months 77=Uncertain

Outcome: ***QN: When you contacted [Name of the person/ places] would you say you felt.... in regard to your symptoms? [Prompt with responses]***
01 = Better 02 = Same 03 = Worse 77=Uncertain

Date of Interview ☐☐ / ☐☐ / 2 0 ☐☐ (Day / Month / Year) STUDY ID# ☐☐☐

Post-Diagnosis and Treatment Pathways

Now, I would like to ask you about the time you learned about the diagnosis of TB

27. Did you receive TB medications on the same day of your current diagnosis?

☐ Yes [Skip to Qn 33] ☐ No

28. If "NO" to Qn. 27, What reasons prevented you from getting the medications? Tick all that is applicable

- ☐ Drug stock out
- ☐ Health Facility was closed
- ☐ No time, needed to go to work/some place
- ☐ Faciltiy was open but no health provider
- ☐ Did not know medications were free of charge
- ☐ Didn't have money to pay for service
- ☐ Uncertain
- ☐ Other,Specify ☐

☐ Days ☐ Weeks ☐ Months

29. How long did it take from diagnosis to receiving TB medications?

☐☐ Days ☐☐ Weeks ☐☐ Months

30. Did you receive TB medications at the same place of diagnosis? ☐ Yes ☐ No

31. If "NO" how many places did you have to visit after diagnosis before you got TB medication? ☐☐

32. What health facility did you visit after diagnosis in an attempt to receive TB medications?

	Name of health facility	Code	Time			
Visit 1		☐☐	☐☐	☐ Days	☐ Weeks	☐ Month
Visit 2		☐☐	☐☐	☐ Days	☐ Weeks	☐ Month
Visit 3		☐☐	☐☐	☐ Days	☐ Weeks	☐ Month

Code for places visited for treatment ***QN: What health facilities did you visit for TB medications?***

01 = Gov't hospital 02 = Private hospital 03 = Private clinic 04 = Gov't Heath center 05=Pharmacy 88=Other (Specify)

33. What is your smoking status? ☐ Current smoker ☐ Previous smoker ☐ Non-smoker

Int Initials:	☐☐	Comp Date:	☐☐ /	☐☐ /	2 0 ☐☐
Rev Initials:	☐☐	Rev Date:	☐☐ /	☐☐ /	2 0 ☐☐
DMO Initials:	☐☐	DMO Date:	☐☐ /	☐☐ /	2 0 ☐☐
CD Initials:	☐☐	CD Date:	☐☐ Day /	☐☐ Month /	2 0 ☐☐ Year
